# Cyberchondria and Health Anxiety in Patients Visiting Hematology Outpatient Clinic: Reflection of Digitalisation

**DOI:** 10.3390/jcm14196795

**Published:** 2025-09-25

**Authors:** Kadir Ilkkilic, Recep Evcen

**Affiliations:** 1 Department of Hematology, School of Medicine, Recep Tayyip Erdogan University, Rize 53200, Turkey; 2 Division of Clinical Immunology and Allergy, Department of Internal Medicine, Recep Tayyip Erdogan University Training and Research Hospital, Rize 53200, Turkey; r_evcen@hotmail.com

**Keywords:** cyberchondria, health anxiety, hematology

## Abstract

**Background:** Cyberchondria is characterized by heightened health anxiety resulting from excessive online health information seeking, and studies on this topic in the field of hematology are limited. The aim of this study was to examine the levels of cyberchondria and health anxiety among patients attending the hematology outpatient clinic without a diagnosis of malignancy, and to evaluate the relationship between these two factors. **Methods:** This prospective cross-sectional study was conducted at the hematology outpatient clinic of Recep Tayyip Erdogan University School of Medicine in Rize, Turkey. The 400 patients included in the study were divided into groups according to their reasons for visiting the outpatient clinic: hemoglobin disorders, leukocyte disorders, and platelet disorders. The severity of cyberchondria was assessed using the Cyberchondria Severity Scale-12 (CSS-12), and health anxiety level was assessed using the Short Health Anxiety Inventory (SHAI). **Results:** The mean age of the 400 patients (255 female, 145 male) was 37.7 ± 11.2 years (18–60 years). The mean SHAI score for patients was 16.1 ± 6.6, and the mean CSS-12 score was 28.7 ± 7.4. Patients presenting with platelet disorders had the highest SHAI scores (18.4 ± 5.6), followed by patients presenting with leukocyte disorders (16.7 ± 6.4) and hemoglobin disorders (15.5 ± 6.8) (*p* = 0.009). In terms of CSS-12 scores, the highest values were found in patients presenting with leukocyte disorders (31.8 ± 8.5), followed by platelet disorders (30.1 ± 7.7) and hemoglobin disorders (27.6 ± 6.7) (*p* < 0.001). There was a positive relationship between health anxiety level and the severity of cyberchondria (r = 0.413, *p* < 0.001) **Conclusions:** The positive correlation observed between cyberchondria severity and health anxiety level underscores the need to consider psychological effects in hematology patients. This clinical condition may increase the burden of disease and should not be overlooked by physicians.

## 1. Introduction

With the rapid advancement of digitalization in healthcare, patients are increasingly using the internet to obtain health-related information. This trend has contributed to heightened health anxiety, characterized by excessive and unrealistic concerns about actual or potential illnesses, and has brought cyberchondria, which arises from repeated online health searches, to the forefront [1].

Increased health literacy through internet searches can contribute positively to health-related decision-making, but it may also give rise to certain problems [2]. Some individuals may feel more anxious after conducting online health research, while others may develop a positive decision-making behavior. Uncertain and contradictory information obtained in the digital environment can increase anxiety levels in patients. Health anxiety is characterized by excessive, persistent, and disproportionate worry about having a serious illness, and it can negatively affect social, occupational, and daily functioning [3]. One study examining individuals’ tendencies to self-diagnose online and investigate their medical concerns found that these activities led to an increase in anxiety levels in two out of five participants [4].

Cyberchondria refers to heightened health concerns and anxiety resulting from repeatedly searching for health-related information online, and the term has come to describe the negative consequences associated with such online health information seeking [5]. It is considered a multidimensional condition encompassing key characteristics such as excessive online searching (excessiveness), the impact of online searching on other activities in daily life (compulsiveness), high anxiety caused by online searching (distress), and seeking professional advice to confirm the accuracy of information found online (assurance) [6].

The severity of cyberchondria has been previously investigated in conditions such as allergic diseases, metabolic syndrome, and urological diseases, but no study has been reported in the context of hematological diseases [7,8,9]. Patients visiting hematology clinics may encounter serious and chronic illnesses, which can contribute to increased health anxiety. Additionally, patients’ misinterpretation or incomplete understanding of medical information further exacerbates this anxiety [10]. Given the complexity of the diagnosis and treatment processes for hematology patients, the potential for cyberchondria and health anxiety to negatively affect clinical compliance is particularly high in this patient group. In this context, the present study is important for understanding patients’ psychological well-being and guiding physicians in managing patients’ information seeking behaviors and health-related anxiety.

The aim of this study was to assess the levels of cyberchondria and health anxiety among patients attending the hematology outpatient clinic without a diagnosis of malignancy, and to evaluate the relationship between these two factors.

## 2. Materials and Methods

### 2.1. Study Design and Participants

This prospective cross-sectional study was conducted between January and June 2025 at the hematology outpatient clinic of Recep Tayyip Erdogan University School of Medicine, Rize, Turkey. Ethics committee approval for the study was obtained from the Non-Interventional Clinical Research Ethics Committee of Recep Tayyip Erdogan University Faculty of Medicine. (Approval number: 2024/300 Approval date: 18 December 2024). The study was conducted in accordance with the Declaration of Helsinki. Written informed consent was obtained from all patients participating in the study. Based on the reasons for hospital presentation and laboratory test results, the patients were classified into three groups: hemoglobin disorders (anemia and polycythemia), leukocyte disorders (leukopenia and leukocytosis), and platelet disorders (thrombocytopenia and thrombocytosis).

Inclusion criteria: Patients were eligible if they were between 18 and 60 years of age; had no diagnosed psychiatric or mental illness; were not receiving psychiatric medication; actively using the internet to obtain health-related information; and had no history of malignancy.

Exclusion criteria: Patients were excluded if they had a history of cancer, any diagnosed psychiatric disorder or were receiving psychiatric treatment, failed to complete the questionnaire, declined to participate, did not use the internet for health-related information, or were older than 60 years.

### 2.2. Data Collection and Measures

Data were collected using two standardized instruments: the Cyberchondria Severity Scale (CSS-12) and the Short Health Anxiety Inventory (SHAI). In addition, patients’ sociodemographic characteristics, including age, gender, marital status, educational level, and presence of chronic diseases, were recorded.

### 2.3. Cyberchondria Severity Scale (CSS-12)

The Cyberchondria Severity Scale consists of two versions: the original 33-item form Cyberchondria Severity Scale (CSS-33) and the short form with 12 items Cyberchondria Severity Scale (CSS-12). CSS-33 is a self-assessment scale developed to measure anxiety and behavioral effects associated with excessive online health research. It is a five-factor psychometric measure consisting of 33 items designed to assess the multidimensional structure of cyberchondria (Compulsion, Distress, Excessiveness, Assurance Seeking, Distrust of Medical Professionals) [6].

In the present study, the CSS-12 was used. This self-report scale comprises 12 Likert-type items scored from 1 (“never”) to 5 (“always”), with higher scores indicating greater severity of cyberchondria [11]. Although no standardized cut-off value exists, higher total scores reflect more pronounced symptoms. The Turkish adaptation, validated in adult and working populations, demonstrated a four-factor structure and good internal consistency (Cronbach’s α = 0.80) [12].

### 2.4. The Short Health Anxiety Inventory (SHAI)

The Short Health Anxiety Inventory (SHAI), developed by Salkovskis et al., is an 18-item self-report instrument designed to evaluate the severity of health anxiety [13]. Each item is rated on a 4-point Likert scale, with higher total scores reflecting greater levels of health anxiety. In the present study, health anxiety was measured using the validated Turkish version of the SHAI, which has demonstrated excellent internal consistency (Cronbach’s α = 0.91), satisfactory item–total correlations, and acceptable test–retest reliability in Turkish populations [14].

### 2.5. Statistical Analysis

All statistical analyses were performed using SPSS software (version 22.0; IBM Corp., Armonk, NY, USA). A priori power analysis was performed using G*Power 3.1. For one-way ANOVA with five groups (α = 0.05, 1 − β = 0.80), an observed omnibus effect (F ≈ 11–12) corresponds to a medium-to-large effect size (f ≈ 0.35). The required total sample size was approximately *n* = 105, which was well below the achieved sample size of *n* = 400, thus ensuring sufficient statistical power [9]. The normality of continuous variables was assessed using the Kolmogorov–Smirnov test, skewness and kurtosis values (within the ±1 range), and visual inspections of histograms and Q–Q plots. Although the Kolmogorov–Smirnov test showed *p* < 0.05, the data were considered to be normally distributed in accordance with the central limit theorem given the large sample size (*n* = 400) and the acceptable skewness/kurtosis values limits. Accordingly, the independent samples *t*-test was used to compare two groups, while one-way ANOVA (F-test) was used for comparisons among three or more groups. The relationships between age, health anxiety (SHAI), and cyberchondria severity (CSS-12), as well as the relationship between health anxiety and cyberchondria severity, were evaluated using Pearson correlation analysis. A *p* < 0.05 value was considered statistically significant.

## 3. Results

A total of 400 patients were included in this study, of whom 255 (64%) were female and 145 (36%) were male. The mean age of the patients was 37.7 ± 11.2 years, with a range of 18–60 years.

Of the patients, 273 (68%) were classified into the hemoglobin disorders group, 73 (18%) into the leukocyte disorders group, and 54 (14%) into the platelet disorders group.

The mean Short Health Anxiety Inventory (SHAI) score of patients was 16.1 ± 6.6, and the mean Cyberchondria Severity Scale-12 (CSS-12) score was 28.7 ± 7.4.

In comparisons by gender, the mean SHAI score for women was found to be significantly higher than that for men (women: 16.9 ± 6.8; men: 14.7 ± 6.0; *p* = 0.001). However, no significant difference was found between genders in CSS-12 scores (*p* = 0.797).

When evaluated according to the reasons for admission, significant differences were observed in both SHAI and CSS-12 scores. Patients presenting with platelet disorders had the highest mean SHAI scores (18.4 ± 5.6), followed by those with leukocyte disorders (16.7 ± 6.4) and hemoglobin disorders (15.5 ± 6.8) (*p* = 0.009). In terms of CSS-12 scores, the highest mean values were observed among patients with leukocyte disorders (31.8 ± 8.5), followed by those with platelet disorders (30.1 ± 7.7) and hemoglobin disorders (27.6 ± 6.7) (*p* < 0.001) (Table 1).

Post hoc analyses revealed that patients with platelet disorders had significantly higher SHAI scores than those with hemoglobin disorders (*p* = 0.009). Regarding CSS-12 scores, patients with leukocyte disorders had significantly higher scores than those with hemoglobin disorders (*p* < 0.001) (Table 2).

No significant differences in SHAI and CSS-12 scores were found with respect to educational level, marital status, or the presence of chronic diseases.

**Figure 1:**
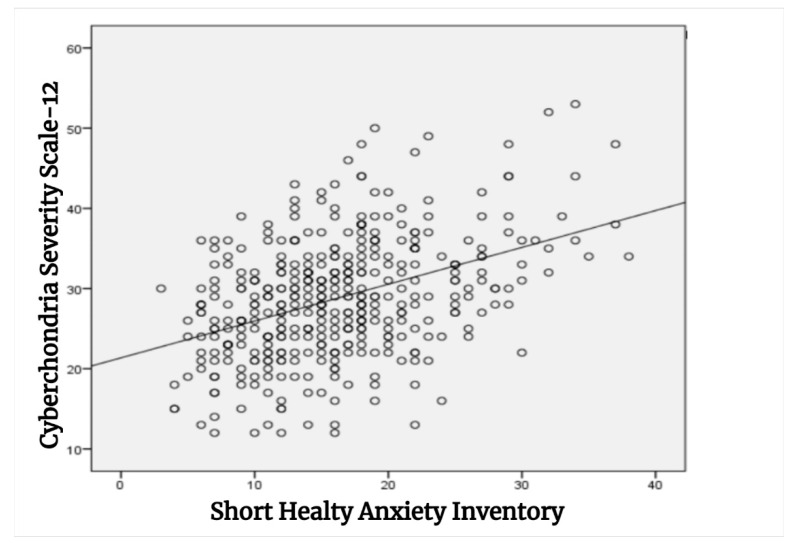
Correlation analysis between the Cyberchondria Severity Scale-12 and the Short Health Anxiety Inventory.

Finally, Pearson correlation analysis showed a moderate positive and statistically significant relationship between SHAI and CSS-12 scores (r = 0.413, *p* < 0.001) (Figure 1).

## 4. Discussion

This study is the first to assess the severity of cyberchondria and the levels of health anxiety among patients attending a hematology outpatient clinic without a malignancy diagnosis. The highest CSS-12 scores were observed in patients presenting with leukocyte disorders, while the highest SHAI scores were found in patients with platelet disorders. In contrast, both CSS-12 and SHAI scores were lowest among patients presenting with hemoglobin disorders. Moreover, a positive correlation was found between the severity of cyberchondria and the level of health anxiety.

The widespread and largely unrestricted use of the internet for accessing health information brings certain challenges. When searching for health information online, the presentation of multiple potential differential diagnoses—including rare but conditions may provoke anxiety in patients [15]. In addition, inconsistent and unconfirmed information on the internet along with the complexity of medical terminology has been shown to contribute to the development of cyberchondria and health anxiety [5].

Previous studies investigating the effect of gender on health anxiety and cyberchondria severity have reported higher levels of both conditions among women [16,17]. On the other hand, Aulia et al. reported no significant gender differences in cyberchondria severity in their study [18]. In the present study, health anxiety were found to be higher among females, although no gender differences were observed in cyberchondria severity. This finding may be attributed to factors such as heightened sensitivity to stress hormones, a tendency toward persistent negative thinking, greater use of the internet and social media, and stress associated with gender roles.

Evidence from recent studies suggested a positive association between higher levels of education and increased cyberchondria severity. For example, one study reported that individuals with higher education levels exhibited more severe cyberchondria [19], and another found that individuals with a high school education or higher had higher cyberchondria scores [20]. Overall, the literature suggests that cyberchondria severity tends to an increase with educational level. However, no relationship was found between patients’ education level and cyberchondria severity in the present study. This may be due to variations in health literacy related to cultural differences or differences in sample characteristics.

The relationship between chronic illness and cyberchondria has been evaluated in previous studies, yielding varying results. In a study by Gergin et al., it was shown that chronic illnesses do not affect levels of cyberchondria [21]. In contrast, a study conducted during the COVID-19 pandemic reported that students with chronic illnesses had higher levels of health anxiety [22]. In the present study, there was no relationship between cyberchondria severity and the presence of chronic illness. This may be explained by the fact that individuals with chronic illnesses experienced a more severe course of COVID-19 and exhibited higher anxiety levels during the pandemic.

The literature shows mixed findings regarding the relationship between age and cyberchondria. A study evaluating the relationship between health literacy and cyberchondria found that younger individuals experienced higher levels of cyberchondria [23]. On the other hand, a study by Latoo et al. found no relationship between age and severity of cyberchondria [24]. This study found no significant association between age and cyberchondria. A study conducted on COVID-19 patients showed no correlation between age and health anxiety [25]. On the other hand, a study by Santoro et al. found a negative association between age and health anxiety [26]. The present study identified a weak but significant negative correlation between age and SHAI scores. This difference may stem from differences in sample characteristics, cultural contexts, or the timing of measurements during the pandemic.

Cultural factors can have significant effects on the development of cyberchondria. Recent studies have shown that health literacy, health beliefs, social relationships, and internet access significantly affect individuals’ levels of cyberchondria [27,28]. For example, in some societies, habits such as consulting family members before visiting a doctor or orientation towards traditional medicine may affect the development of cyberchondria [19]. Our findings should be evaluated considering Turkey’s cultural structure.

Visits to hematology outpatient clinics due to leukocyte or platelet disorders are relatively common. Abnormalities in blood values carry varying prognoses and potential health threats, which can elicit different levels of emotional distress in patients. Prior exposure to unclear, contradictory and outdated online information may exacerbate patients’ health anxiety. A study has shown that patients who search the internet for health information tend to focus on more serious medical conditions rather than more common, benign causes [29]. This condition becomes more pronounced in the case of serious and fatal diseases that can manifest with leukocyte and platelet disorders, such as acute leukemia [30]. Culturally, acute leukemia is referred to as “blood cancer” in our society, and online searches about the disease can significantly amplify health anxiety. In this context, one possible reason for the higher levels of health anxiety in leukocyte disorders and the higher severity of cyberchondria in platelet disorders found in the current study may be this condition.

In a study conducted by Blackburn et al., orthopedic patients who sought medical information online exhibited less tolerance for uncertainty, developed more health-related concerns, and showed increased cyberchondria severity [31]. Similarly, a study conducted in a urology clinic also found that cyberchondria was more pronounced among patients with andrological and uro-oncological diseases, accompanied by higher health anxiety levels [9]. Our current results are consistent with these studies previously conducted in different clinics. This consistency may be related to the use of the same measurement tools and the fact that the studies were conducted in similar clinical settings.

Recent studies have highlighted the vital role of platelets and leukocytes in inflammatory responses, confirming their importance in chronic inflammatory diseases by supporting the formation and development of inflammation [32,33]. A study conducted by Dregan et al. found that the prevalence of depression and anxiety was higher in patients with inflammatory disorders [34]. Similarly, a study on children and adolescents suggested a possible link between chronic inflammation and anxiety disorder based on hemogram parameters [35]. During online searches for causes of leukocyte or platelet disorders, patients may encounter information on chronic inflammatory diseases that are persistent, recurrent and difficult to fully resolve. This condition, combined with the uncertainty that persists until a definitive diagnosis is made, may contribute to increased health anxiety and levels of cyberchondria in patients.

A study investigating the role of hemoglobin levels in anxiety found no clear evidence of a relationship between anxiety and hemoglobin levels [36]. Another study found that low baseline hemoglobin levels were strongly associated with depression in older men but not in women [37]. In the present study, both cyberchondria and health anxiety scores were found to be lowest in patients presenting with hemoglobin disorders. This may be because hemoglobin disorders are more common, yet patients tend to associate them less with cancer compared to leukocyte and platelet disorders.

Previous studies have consistently demonstrated a strong relationship and overlap between cyberchondria and health anxiety [38,39]. Consistent with these findings, present study demonstrated a positive correlation between cyberchondria severity and health anxiety levels. This suggests that intense and uncontrolled searches for health-related information may fuel health anxiety and increase their anxiety levels.

### 4.1. Strengths and Limitations

This study is the first to evaluate the severity of cyberchondria and the level of health anxiety in patients attending a hematology outpatient clinic for leukocyte, platelet, or hemoglobin disorders who did not have a malignancy diagnosis, providing important insights.

However, the study had several limitations. The sample size was relatively small, and the research was conducted at a single center. Another limitation is the use of the 12-item short form (CSS-12) instead of the original 33-item CSS-33. Although the validity and reliability of CSS-12 in Turkish have been demonstrated and it offers advantages in terms of practical applicability, it may not capture the multidimensionality of cyberchondria as thoroughly as CSS-33. Additionally, the cross-sectional design of this study allows for correlation analysis but does not support causal inferences. Potential biases from data collection and self-reporting may also limit the extent to which the results reflect patients’ actual behavior. Current limitations may restrict the generalizability of the study’s results.

### 4.2. Suggestions for Further Research

In the present study, we evaluated the severity of cyberchondria and health anxiety levels in hematology outpatient clinic patients who actively use the internet for health information, highlighting the significant impact of an increased severity of cyberchondria and health anxiety in individuals with leukocyte–platelet disorders. However, we did not specifically evaluate the relationship between these conditions and the use of artificial intelligence (AI). The use of artificial intelligence in obtaining health information has been rapidly increasing in recent years, yet we did not identify any specific studies investigating whether AI use in the context of hematological diseases affects cyberchondria or health anxiety. Further research could explore the effect of artificial intelligence use on cyberchondria and health anxiety levels in hematological diseases, allowing for the implementation of measures to alleviate patient and physician concerns from both psychosocial and clinical perspectives, thereby reducing the burden of disease.

## 5. Conclusions

Cyberchondria may influence the patient’s decisions regarding diagnosis, treatment and overall health management in the hematology outpatient clinic. The positive correlation observed between cyberchondria severity and health anxiety level underscores the need to consider psychological effects in hematology patients. This clinical condition should not be overlooked by physicians. In order to minimize health anxiety and cyberchondria severity, patients should receive proper guidance on responsible internet use and access to reliable health information. When appropriate, referrals to cognitive behavioral therapy may also help reduce the psychological and clinical burden of illness.

## Figures and Tables

**Table 1:** Sociodemographic characteristics of patients and comparison of CSS-12 and SHAI scores.

		SHAI	*p* Value	CSS-12	*p* Value
	*n* (%)	Mean ± SD		Mean ± SD	
Gender					
Male	145 (36)	14.7 ± 6.0	0.001	28.9 ± 6.9	0.797
Female	255 (64)	16.9 ± 6.8		28.7 ± 7.6	
Group					
Hemoglobin disorders	273 (68)	15.5 ± 6.8	0.009	27.6 ± 6.7	<0.001
Leukocyte disorders	73 (18)	16.7 ± 6.4		31.8 ± 8.5	
Platelet disorders	54 (14)	18.4 ± 5.6		30.1 ± 7.7	
Education Level					
Primary Education	101 (25)	16.7 ± 7.0	0.556	28.0 ± 7.3	0.508
High School	132 (33)	15.7 ± 6.6		29.1 ± 7.6	
University	167 (42)	16.0 ± 6.4		28.9 ± 7.2	
Marital Status					
Single	117 (29)	16.8 ± 6.5	0.323	28.6 ± 6.8	0.576
Married	268 (67)	15.7 ± 6.7		28.9 ± 7.7	
Widowed	15 (4)	17.0 ± 6.2		26.8 ± 5.7	
Chronic Disease Status					
Yes	122 (30)	16.5 ± 6.3	0.453	28.8 ± 7.1	0.704
No	278 (70)	15.9 ± 6.7		28.5 ± 7.8	

SHAI: Short Health Anxiety Inventory, CSS-12: Cyberchondria Severity Scale (Short-form version).

**Table 2:** Post Hoc Analysis of Groups.

Group	Other Groups	*p* Value * (SHAI)	*p* Value ** (CSS-12)
Hemoglobin disorders	Leukocyte disorders	0.318	<0.001
	Platelet disorders	0.009	0.058
Leukocyte disorders	Hemoglobin disorders	0.318	<0.001
	Platelet disorders	0.350	0.396
Platelet disorders	Hemoglobin disorders	0.009	0.058
	Leukocyte disorders	0.350	0.396

ANOVA test *p* < 0.05, * *p* value between groups: 0.009 (F: 4.762), ** *p* value between groups: <0.001 (F: 10.641). ANOVA: Analysis of Variance. SHAI: Short Health Anxiety Inventory, CSS-12: Cyberchondria Severity Scale (Short-form version).

## Data Availability

The data are available from the corresponding author upon reasonable request.
